# Nuclear Receptors as Autophagy-Based Antimicrobial Therapeutics

**DOI:** 10.3390/cells9091979

**Published:** 2020-08-27

**Authors:** Prashanta Silwal, Seungwha Paik, Sang Min Jeon, Eun-Kyeong Jo

**Affiliations:** 1 Department of Microbiology, Chungnam National University School of Medicine, Daejeon 35015, Korea; pst.ktz@gmail.com (P.S.); swpaik@gmail.com (S.P.); valersangmin@gmail.com (S.M.J.); 2 Infection Control Convergence Research Center, Chungnam National University School of Medicine, Daejeon 35015, Korea

**Keywords:** nuclear receptors, autophagy, infections, host defense

## Abstract

Autophagy is an intracellular process that targets intracellular pathogens for lysosomal degradation. Autophagy is tightly controlled at transcriptional and post-translational levels. Nuclear receptors (NRs) are a family of transcriptional factors that regulate the expression of gene sets involved in, for example, metabolic and immune homeostasis. Several NRs show promise as host-directed anti-infectives through the modulation of autophagy activities by their natural ligands or small molecules (agonists/antagonists). Here, we review the roles and mechanisms of NRs (vitamin D receptors, estrogen receptors, estrogen-related receptors, and peroxisome proliferator-activated receptors) in linking immunity and autophagy during infection. We also discuss the potential of emerging NRs (REV-ERBs, retinoic acid receptors, retinoic acid-related orphan receptors, liver X receptors, farnesoid X receptors, and thyroid hormone receptors) as candidate antimicrobials. The identification of novel roles and mechanisms for NRs will enable the development of autophagy-adjunctive therapeutics for emerging and re-emerging infectious diseases.

## 1. Introduction

Autophagy, an intracellular process, is a defense against intracellular pathogens involving lysosomal degradation [1,2,3,4,5,6]. The signaling factors and mechanisms through which invading microbes are selectively targeted by xenophagy or LC3-associated phagocytosis (LAP) have been reported [7]. A variety of pathogens—including *Mycobacterium tuberculosis* (Mtb), *Salmonella enterica serovar Typhimurium*, *Listeria monocytogenes*, *Legionella pneumophila*, *Anaplasma phagocytophilum*, *Coxiella burnetii*, *Francisella tularensis*, and *Brucella spp.*—can be targeted by autophagy [7]. These and other pathogens, including viruses and protozoa, have evolved means of evading or circumventing autophagy, enabling their replication within host cells [5,6,8,9,10,11,12,13,14].

The nuclear receptor (NR) superfamily proteins regulate genes involved in cell survival, signaling, metabolism, and reproduction [15,16,17]. NRs promote host defense against infections by regulating innate immunity, the transcription of antimicrobial genes, and signaling pathways [18,19]. NRs are implicated in the regulation of autophagy at transcriptional and post-translational levels [17,20,21,22,23]. Modulating NR activity by targeting the NR domains or by promoting ligand activation/suppression, modulation of the NR-DNA interaction, and/or the recruitment of coactivators may be effective against, for example, cancer, metabolic and immune diseases, inflammation, and neurodegeneration [17,24,25,26,27].

The interaction of host autophagy with pathogens has been reviewed by others [28,29,30,31]. Here, we discuss the roles of autophagy-related genes (ATGs) in the immune response to pathogens and how various NR ligand-based approaches are being implemented for antimicrobial modalities based on autophagy activation.

## 2. Overview of Autophagy and Autophagy-Related Genes

A series of ATGs play critical roles in autophagy initiation, elongation, and maturation, and are also involved in other physiological responses, including membrane trafficking and signaling pathways [32,33,34,35]. ATG proteins in complexes with other cofactors/regulators modulate autophagy [36]. However, unlike in canonical autophagy, non-canonical autophagy does not necessarily require double-membraned autophagosome formation involving canonical initiation, nucleation, and an elongation step [37]. For example, a double-membraned autophagosome constituted from multiple isolated membranes is found in some cases of xenophagy, and even a single-membrane bound phagosome is formed in LAP [37]. Therefore, distinct subsets of ATGs are required for the activation of non-canonical autophagy LAP [38] or selective autophagy [34].

The roles of ATGs differ according to the type of autophagy. Rubicon plays opposite roles in (macro)autophagy and LAP; it is a negative regulator of autophagy, but promotes Beclin-1/VPS34 kinase activity in the phagosome [34,39]. Selective autophagy receptors have both a cargo recognition function and interact with the autophagosome [40,41]. The selective autophagy receptor p62/AQSTM1 plays several roles in numerous biological processes [42,43]. During nonselective autophagy, p62 degradation reflects activation of the autophagic flux, as the cargo receptor p62 is essential for selective autophagy [41]. Therefore, the overlapping cooperative and antagonistic roles of ATGs in autophagy need to be taken into account when developing host-directed therapeutics [34,44,45].

Model studies using vertebrates (mice) and invertebrates (*Caenorhabditis elegans* and *Drosophila*) and involving tissue-specific ATG deletion or overexpression have provided pathogenetic insight by identifying autophagy-dependent and -independent functions of ATGs [46,47]. Additionally, the dysregulation or mutation of ATGs is associated with diverse human diseases [34]. In response to stresses, ATG upregulation at the transcriptional level induces autophagy in a manner requiring numerous transcription factors and signaling cofactors [48,49,50]. In addition, the effect of ATGs and non-ATG proteins on autophagy is modulated by posttranslational modifications, including phosphorylation, glycosylation, ubiquitination, acetylation, and lipidation [48,51,52,53,54,55].

Antibacterial autophagy modulates bacterial replication and promotes innate immunity in host cells. Increasing evidence has shown that various intracellular bacteria, such as Mtb, *Salmonella*, *Listeria*, and *Legionella*, could be targeted by autophagy activation [7,56]. Autophagy not only directly causes microbial degradation, but also functions as a host defense system by participating in intracellular signaling, lysozyme secretion, the ubiquitin pathway, and antigen presentation [4]. However, these pathogens have also evolved strategies to evade or subvert host autophagy to survive within host cells, resulting in persistent infection and pathogenesis [57]. Similarly, autophagy is one of the key defense mechanisms in protecting against viral infections [58]. It regulates the immune response through the selective degradation of immune components associated with viral particles, followed by virus-derived antigen presentation to T lymphocytes to coordinate adaptive immunity. However, viruses manipulate and exploit autophagy for their immune evasion, replication, and release from host cells [59,60]. It is surprising that even if viruses target similar host defense pathways during the infection state, they differ in ways and functional outcomes, depending on each species of virus [59]. In order to increase the understanding on the role of autophagy dominating viral infections, not only common pathways, but also various species-specific studies, should be conducted in parallel. So far, the exact functions of ATG proteins and the detailed mechanisms that control autophagy during viral infection have not been elucidated [59].

In this review, we focus on the pathophysiological roles and mechanisms underlying the NR-mediated regulation of autophagy, including the activation of antimicrobial responses in various infection models. 

## 3. Overview of Nuclear Receptors

**Figure 1:**
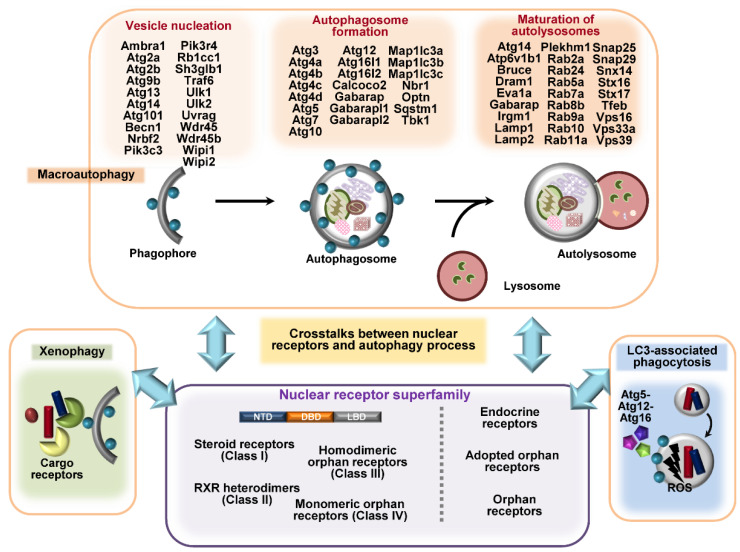
A summarized figure for autophagy genes and different classes of nuclear receptors (NRs). Autophagy processes such as macroautophagy, LC3-associated phagocytosis (LAP), and xenophagy involve different autophagy-related genes (ATGs) or cargo receptors, such as p62, NDP52, and optineurin. The upper panel highlights the different sets of autophagy genes involved in vesicle nucleation, autophagosome formation, and the maturation of autolysosomes. The NR superfamily classes are divided into three or four subclasses according to their structural and functional characteristics and their ligands. NRs are implicated in the regulation of autophagy at transcriptional and post-translational levels. Understanding the mechanisms by which NRs regulate the expression and post-translational modification of ATGs will facilitate the development of novel host-directed antimicrobial agents.

The NR superfamily classes are divided into four classes based on structural and functional characteristics, i.e., steroid receptors (Class I), retinoid X receptor (RXR) heterodimers (Class II), homodimeric orphan receptors (Class III), and monomeric orphan receptors (Class IV) [15,61]. The NR superfamily is classified as the endocrine, adopted orphan, and orphan subfamilies, depending on the existence of ligands [61] (Figure 1). The differences in NR classes include their biological function, binding to a ligand or DNA, and tissue specificity. All members of the NR superfamily have a variable N-terminal domain (NTD), a DNA binding domain (DBD), a ligand-binding domain (LBD), and a variable C-terminal domain. NR DBD contains different DNA-binding recognition sequences and two zinc finger motifs for binding to chromatin [61,62]. NRs play a crucial role in the recruitment of co-activators within the nucleus, although there is marked variability among the binding of NTDs to co-activators [15].

A total of 48 intracellular proteins have been identified as NRs [63]; among them, several members are critical in the regulation of host immune responses to infection. These NRs include the vitamin D receptor (VDR), also known as nuclear receptor subfamily 1, group I, member 1 (NR1I1) [64,65,66]; estrogen-related receptor-α (ERRα; ESRRA; NR3B1) [20,67]; ERRγ (ESRRG; NR3B3) [68]; liver X receptor-α (LXRα; LXRA; NR1H3) [69]; peroxisome proliferator-activated receptor-α (PPARα; PPARA; NR1C1) [70,71,72]; PPARγ (PPARG; NR1C3) [73,74,75]; the glucocorticoid receptor (GR; GCR; NR3C1) [76]; estrogen receptor-α (ERα; ESR1; NR3A); ERβ (ESR2; NR3A2) [77,78]; the xenobiotic pregnane X receptor (PXR; NR1I2) [79,80,81]; rev-Erb-α (REV-ERBα; NR1D1) [82,83]; farnesoid X receptors (FXRs; NR1H4) [75,84]; nuclear receptor 4A (NR4A) family members; nuclear receptor related 1 protein (NURR1; NR4A2); and neuron-derived orphan receptor 1 (NOR1; NR4A3) [85]. The NRs are endogenously activated by small lipophilic ligands, such as steroid hormones, retinoids, and phospholipids; however, some of the NRs have been classified as ‘orphan’ members as their ligands have not yet been identified [86]. The ligands can cross the plasma membrane, directly interact with NRs inside the cells, and modulate gene transcription through different mechanisms [87].

In class I NRs or steroid receptors, ligand binding at the plasma membrane is followed by a signal transduction cascade including enzymatic phosphorylation, which results in the translocation of transcription factors into the nucleus [61,88]. ER, a member of the class I NR superfamily, is anchored in the cytoplasm by a chaperone protein such as heat shock protein 90 (HSP90). After ligand binding, the receptor is freed from the chaperone, causing homodimerization and nuclear translocation. In the nucleus, the ligand-receptor complex associates with the transcriptional coactivator and activates the target gene [89]. Selective estrogen receptor modulators (SERM) such as tamoxifen and bazedoxifen have been suggested to have antimycobacterial activity [90,91].

The class II receptor family includes the thyroid hormone receptor (TR), VDR, retinoic acid receptor (RAR), and PPAR [61]. They are typically present in the nucleus and generally form heterodimers with RXR [92]. The heterodimers are bound to their response element, even in the absence of a ligand, where gene activation is repressed through interaction with a nuclear co-repressor (NCoR) and silencing mediator for retinoic acid and thyroid hormone receptor (SMRT) corepressor complexes. Binding of the ligand causes displacement of the NCoR/SMRT co-repressor, allowing transcriptional activation to occur [89,93]. For VDR, 1α,25-dihydroxyvitamin D3 (1,25(OH)2D3), which is the active form of vitamin D3 (hereafter referred to as vitamin D), acts as a ligand and activates functional VDR, which then recognizes and binds to vitamin D response elements (VDREs) located in the promoter region of target genes to control the transcription of those genes [94]. VDR signaling activation during infection leads to innate immune signals for the production of antimicrobial peptides (AMPs), such as human cathelicidin AMP (CAMP) and β-defensin 2, which are important in coordinating vitamin D-induced antimicrobial responses [95]. PPARs include three different isotypes: PPARα, PPARβ/δ (PPARD; NR1C2), and PPARγ. Each isoform has a different distribution and ligands [96]. They are activated after the binding of endogenous ligands, such as fatty acids and their derivatives, or synthetic modulators, such as GW7647 and GW501516, to the ligand-binding domain. PPARs form heterodimers with RXR, which, after ligand binding, results in the transactivation or repression of target genes through PPAR responsive elements (PPREs) [96,97]. LXRs are the NRs which bind oxidized cholesterol derivatives such as oxysterols and intermediates of the cholesterol biosynthesis pathway [98]. The modulation of gene expression by LXR involves direct activation, repression, and transrepression [99], and exhibits anti-inflammatory properties, as well as antimicrobial effects [98]. 

**Figure 2:**
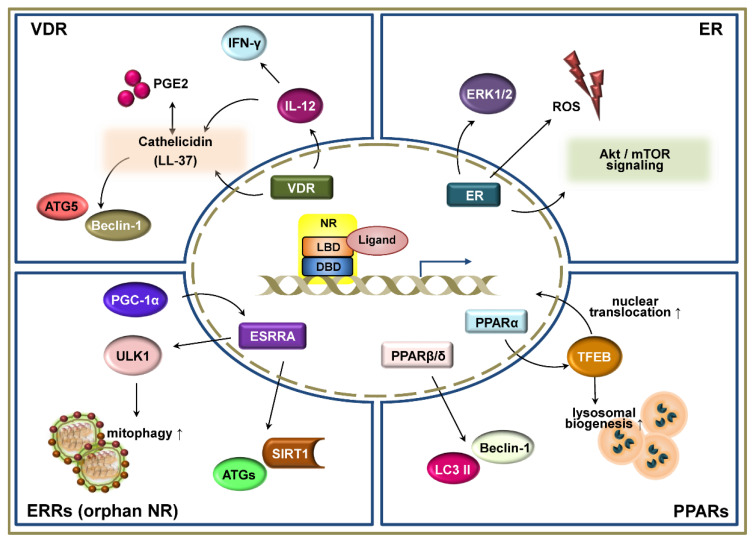
Schematic representation of the signaling pathways of nuclear receptors (NRs) in autophagy-mediated host defense. NRs, including the vitamin D receptor (VDR), estrogen receptor (ER), estrogen-related receptors (ERRs), and peroxisome proliferator-activated receptors (PPARs) have been shown to play critical functions in the regulation of autophagy-mediated host defensive immune responses during infection. These NRs regulate and participate in the autophagic signaling pathways not only at the transcriptional level, but also at the post-transcriptional level. VDR is one of the best characterized NRs related to autophagic function against various infections. It is well-known that VDR signaling increases autophagy activation via the induction of cathelicidin, which is a small cationic antimicrobial peptide. In addition, VDRs functionally link adaptive and innate immune responses by regulating downstream pathways of autophagy. ER activates autophagy by increasing reactive oxygen species (ROS) generation and Akt/ mammalian target of rapamycin (mTOR) signaling. ERRs, which are one of the orphan family members of NR, also regulate a variety of cellular responses, including autophagy. The induction of PGC-1α upregulates the ERRα to promote mitophagy and an antimicrobial effect through sirtuin 1. PPARα activation leads to the expression of transcription factor EB (TFEB) and its nuclear translocation, resulting in the enhancement of lysosomal biogenesis. PPARβ/δ prevents harmful ER stress by increasing autophagy markers Beclin-1 and LC3 II.

Class III and IV receptors include the dimeric and monomeric orphan receptors, respectively. ERRα, one of the orphan nuclear receptors, is not regulated by the presence of natural ligands, but is regulated by post-transcriptional modifications, such as phosphorylation, sumoylation, or acetylation of the N-terminal domain [23,100], and is essential for antimicrobial host defense [23]. Other members of orphan NR include retinoic acid-related orphan receptors (ROR), whereas FXR and REV-ERB have been classified as adopted orphan receptors [92]. In addition, NRs are important in the regulation of autophagy [17,20,27,101], not only at the level of the transcription of ATGs, but also at the post-transcriptional level, by regulating protein–protein interactions, post-translational modification, and epigenetic mechanisms [21,22,23,76,102]. The contributions of NR modulation to defense against pathogens are beginning to be deciphered. Here, we focus on the roles of VDR, ER, ERR, and PPAR in autophagic host defense against infection and discuss other NRs as links between autophagy and innate immunity during infection (Figure 2). A deeper understanding of NR signaling and its underlying mechanisms will facilitate the development of autophagy-based host-directed anti-infectives.

## 4. Vitamin D Receptor in Autophagy-Mediated Defense against Infection

VDR signaling ameliorates infection and inflammation [66,67,103,104]. Studies on the role of vitamin D in innate immunity have revealed that autophagy enhances phagosomal maturation and lysosomal function, and ameliorates inflammation and antimicrobial protein generation [67,105,106]. A physiological level of the active form of vitamin D (1α,25-dihydroxycholecalciferol) or functional activation of VDR signaling promotes autophagy activation in human monocytes or monocytic cells by inducing the synthesis of cathelicidin—a cationic antimicrobial peptide—which promotes phagosomal maturation in the presence of intracellular Mtb [107,108,109] or *Mycobacterium marinum* [110]. In addition, vitamin D treatment and TLR8-mediated VDR signaling activation enhanced autophagy in human macrophages in a manner dependent on the ATG5 and Beclin-1, thereby inhibiting human immunodeficiency virus (HIV)-1 replication or the co-infection of HIV and Mtb [111,112,113]. Vitamin D-mediated autophagy and cathelicidin expression are negatively regulated by prostaglandin (PG)E2—an arachidonic acid-derived lipid mediator—via E prostanoid (EP)2 and EP4 receptors [114].

Vitamin D supplementation in mice significantly induced VDR, cathelin-related antimicrobial peptide (CRAMP), and LC3B expression, but decreased the collagenase matrix metalloproteinase-1 [115]. A structural equation modeling analysis suggested that vitamin D-mediated autophagy reduces necrosis [115]. Additionally, clinical trials of vitamin D as adjunctive therapy to standard anti-tuberculosis (TB) treatment showed a significant decline in intracellular Mtb growth and the levels of proinflammatory cytokines/chemokines [116,117], but no clear effect on long-term sputum-smear conversion [118]. These data suggest that the vitamin D-autophagy pathway is associated with clinical recovery from TB. Nevertheless, further studies are needed to determine the effects and risks of vitamin D adjunctive therapy, as discussed by others [95,119,120]. We limit the discussion in this review to clinical trials of vitamin D therapy in TB and other infectious diseases.

IFN-γ, which is an important cytokine in the adaptive Th1 immune response, alone [121] or in combination with the CD40 ligand [122], enhanced VDR-mediated antimicrobial defense in human monocytes/macrophages in vitamin D-sufficient serum. Vitamin D treatment was required for the expression of IL-12 [123], and the combination of IL-12 and IL-18 in human macrophages enhanced the cell-autonomous production of IFN-γ and the autophagy-cathelicidin pathway, which upregulated the antimicrobial response to Mtb [124]. Therefore, functional VDR signaling links the adaptive and innate immune responses by regulating autophagy, phagosome-lysosome fusion, and cytokine production in Mtb infection.

Vitamin D treatment reversed the influenza A virus-induced inhibition of autophagic flux by inducing the expression of syntaxin-17 and the V-type proton ATPase subunit (ATP6V0A2) [125]. In addition, probiotic lactic-acid bacteria isolated from kimchi activated VDR-autophagy responses and enhanced the expression of ATG16L1 and Beclin-1, resulting in an anti-inflammatory and anti-infective effect in the intestines [126]. Vitamin D treatment restored the lysosomal function impaired by *Helicobacter pylori* in gastric epithelial cells, by activating— protein disulfide isomerase family A member 3 (PDIA3) receptor and upregulating mucolipin-3 (MCOLN3)-mediated Ca^2+^ release [106]. In an animal study of *Aspergillus fumigatus* infection, vitamin D treatment led to autophagic homeostasis by reducing the number of autophagy-mediated lysosomes and regulatory T-cells, thus enhancing the antimicrobial response [127]. Moreover, vitamin D suppressed rotavirus infection by upregulating the autophagy gene Beclin-1 and promoting autophagic maturation and cathelicidin gene expression [128]. Although vitamin D-induced autophagy is critical for an effective immune response, further studies are needed to determine the ability of vitamin D to prevent and treat infectious diseases in humans. The studies on VDR-related autophagy during infection are summarized in Table 1.

## 5. Estrogen Receptors

Estrogen—a female sex steroid hormone—and its receptors (ERα and ERβ) reportedly modulate autophagy, which is implicated in various human diseases and the determination of cell fate [21,129]. Indeed, ERs and the downstream genomic and non-genomic signaling cascades affect the outcomes of tumorigenesis and angiogenesis in breast cancer [21,130]. In addition, ERα activation by estrogen enhances autophagy and tumor cell survival in papillary thyroid cancer by promoting reactive oxygen species (ROS) generation and the activation of extracellular signal-regulated kinases [131].

Estrogen modulators influence antimicrobial responses by influencing autophagy. The selective ER modulator bazedoxifene suppresses the intracellular growth of Mtb by activating autophagy via ROS generation and Akt/ mammalian target of rapamycin (mTOR) signaling [90]. Tamoxifen (TAM) is a potent inhibitor of Shiga toxin trafficking and toxicity in a manner independent on ERs [132]. TAM also restricted Toxoplasma replication by inducing xenophagy or autophagy [133]. Long-term treatment with 17β-estradiol (E2) exerted a beneficial effect on endotoxemia-associated circulatory and multiple organ dysfunction in ovariectomized rats, which was mediated, at least in part, by autophagy activation [134]. Clarification of the mechanisms through which ERs modulate autophagy and/or host defense, as well as the crosstalk between autophagy and immunity in the context of ER signaling, is needed for the development of novel therapeutic modalities for infection and inflammation.

## 6. Estrogen-Related Receptors

ERRs are orphan members of the NR family, and are involved in a variety of biological responses, including cellular metabolism and energy control [135,136,137]. ERRα is a critical regulator of autophagy at transcriptional and post-translational levels, particularly in cooperation with sirtuin 1. These effects promoted the antimicrobial response to Mtb [23]. The thyroid hormone upregulated the expression of ERRα by inducing PGC-1α (PPARGC1A), thus modulating mitochondrial biogenesis and mitophagy. Mechanistically, the thyroid hormone upregulated the autophagy-regulating kinase ULK1 through ERRα, which was required for the autophagic clearance of mitochondria, i.e., mitophagy [138]. In contrast, the inhibition of ERRα activity by the inverse agonist XCT790 induced autophagy and promoted the clearance of toxic protein aggregates, enhancing its neuroprotective effect [139]. There is a role for ERRα in host defense between intracellular bacteria and viruses [23,140,141,142]. Therefore, future studies should clarify the role of ERRα in modulating autophagy and evaluate its therapeutic potential as an antimicrobial. The studies on ERs and ERRα-related autophagy during infection are summarized in Table 2.

## 7. Peroxisome Proliferator-Activated Receptors

### 7.1. Peroxisome Proliferator-Activated Receptor-α

The nutrient-sensing NRs, PPARα and FXR, play reciprocal functions in the regulation of autophagy; PPARα enhances and FXR suppresses autophagy to enhance lipolysis [22] and ciliogenesis [143]. PPARα ameliorates inflammatory and injurious conditions by inducing autophagy in various cells and tissues [144,145]. In addition, autophagy activation leads to PPARα activation by degrading nuclear receptor co-repressor 1 (NCoR1), which interacts with and suppresses the transactivation of PPARα [146].

PPARα modulates antimicrobial responses to Mtb, *Mycobacterium bovis* bacillus Calmette-Guérin (BCG), or *Mycobacterium abscessus* by activating transcription factor EB (TFEB) [70,71,72]. In addition, PPARα deficiency resulted in an exaggerated inflammatory response to mycobacterial infection. Importantly, PPARα activation significantly reduced the lipid body number and size in macrophages infected with Mtb or *M. bovis* BCG, suggesting that PPARα contributes to lipid catabolism and reduces the foamy refuge during mycobacterial infection [72]. TFEB controlled the inflammatory response of host macrophages to Mtb or BCG infection [72]. However, TFEB was reportedly required for the induction of inflammatory cytokines and chemokines in macrophages infected with *Staphylococcus aureus* [147].

Numerous agents have been reported to activate PPARα and enhance TFEB, thereby promoting lysosomal biogenesis in models of chronic inflammatory and degenerative diseases [148,149,150,151]. Therefore, PPARα-activating drugs have potential for various infectious diseases. HIV infection inhibited autophagy in macrophages, promoting the intracellular survival of Mtb and non-tuberculous mycobacteria (NTM) [152]. Trehalose, which targets TFEB and PGC-1α [153], activated the xenophagic flux to eradicate intracellular Mtb and NTM [152]. Mechanistically, Trehalose enhanced TFEB nuclear translocation and autophagy activation in an mucolipin 1 (MCOLN1)-dependent manner [152]. Because trehalose promotes the functionally active conformation of the N-terminal domain of the glucocorticoid receptor [154], its antimicrobial effect may be mediated by GR signaling. Therefore, trehalose-induced autophagy may be involved in controlling co-morbidities of HIV and TB infections.

### 7.2. PPARβ/δ and PPARγ

PPARβ/δ inhibits the ER stress induced by palmitate in AC16 cardiomyocytes by inducing expression of the autophagy markers Beclin-1 and LC3 II, thus preventing the harmful cardiac effects of ER stress [155]. The treatment of septic mice with the PPARβ/δ-agonist GW0742 improved long-term survival and protected against multiple organ injury and dysfunction by modulating inflammatory signaling and coagulation [156,157].

Amodiaquine, which is a selective anti-*Plasmodium falciparum* agent, suppresses autophagolysosomal degradation and PPARγ activity [158]. The PPARγ ligand HP24, which is a pyridinecarboxylic acid derivative, ameliorated the pathologic and inflammatory responses induced by *Trypanosoma cruzi* [159]. INT131, which is a novel non-thiazolidinedione and selective PPARγ modulator, has a beneficial anti-inflammatory effect on EcoHIV-infected glial cells and in a mouse model of EcoHIV infection [160]. However, whether PPARβ/δ or PPARγ activation exerts an antimicrobial effect by modulating autophagy is unclear. Further studies are needed to clarify the roles of PPARβ/δ and PPARγ in controlling the host response to infections in the context of autophagy activation. The studies on PPAR-related autophagy during infection are summarized in Table 3.

## 8. Other Nuclear Receptors Potentially Linking Autophagy and Host Defenses

### 8.1. REV-ERBα and REV-ERBβ

The adopted orphan NR—REV-ERBα—is involved in adipogenesis, muscle differentiation, glucose/lipid metabolism, and the circadian rhythm [161,162,163]. REV-ERBα links the circadian rhythm and autophagy, and directly regulates the rhythmic expression of ATGs in zebrafish [164]. The key transcriptional regulators TFEB and TFE3 are required for the expression of REV-ERBα [162], suggesting another link between autophagy and the circadian cycle.

During infection, REV-ERBα activation by GSK4112 exerted an anti-mycobacterial effect in macrophages by enhancing autophagy and lysosomal biogenesis and suppressing IL-10 synthesis [165]. However, the pharmacological activation of REV-ERBα and REV-ERBβ (NR1D2) using agonists inhibited autophagy and lipogenesis, thereby exerting an anticancer effect [163]. Moreover, the lysosomotropic REV-ERBβ ligand (ARN5187) inhibited autophagy and exerted a cytotoxic effect in breast cancer cells [166]. The over-expression of REV-ERBα in skeletal muscle induced mitochondrial activity and respiratory capacity, but repressed autophagy [167]. Therefore, REV-ERBα and REV-ERBβ may play diverse roles in autophagy regulation, depending on the cell type and pathological status. Because the circadian rhythm may be linked to the immune response to infection [168,169,170], further studies should clarify the roles of REV-ERBα and REV-ERBβ in autophagy, the circadian rhythm, and the immune response to bacterial and fungal pathogens.

### 8.2. Retinoic Acid Receptor-α (RARα; RARA; NR1B1), -β (RARβ; RARB; NR1B2), and -γ (RARγ; RARG; NR1B3)

All-trans retinoic acid and/or arsenic trioxide induced autophagy of the oncoprotein promyelocytic leukemia (PML)/RARA, suggesting RARα as a therapeutic target for acute PML [171,172]. In addition, RARα activated autophagy in human primary B cells [173] and various types of cancer cells [174,175]. The downregulation of RARα led to the upregulation of VDR expression in acute myeloid leukemia cells [176]. However, its role in autophagy during the antimicrobial response is unclear. RARα is reportedly a critical regulator of the maturation of monocyte-derived dendritic cells during HIV infection [177], although its relevance to autophagy has not been evaluated.

Similarly, little is known about the role of RARβ in the regulation of autophagy during infection. RARβ has a tumor suppressive function and is involved in cell differentiation and apoptosis. Interestingly, the human papillomavirus type 16 (HPV16) E7 oncoprotein upregulated the mRNA and protein levels of RARβ in cervical cancer cells and in the cervix of K14E7 transgenic mice [178]. During human adenovirus infection, the RARβ mRNA and protein levels were downregulated, but the overexpression of RARβ decreased human adenovirus production [179]. Therefore, RARβ may have therapeutic effects for adenovirus infection, although the autophagy-mediated suppression of infection is unclear in RARβ-induced antiviral and anticancer effects.

### 8.3. Retinoic Acid-Related Orphan-α (RORα; RORA; NR1F1), -β (RORβ; RORB; NR1F2), and -γ (RORγ; RORC; NR1F3)

RORs are important in the regulation of the circadian clock, metabolic homeostasis, and tumorigenesis [180,181]. Although RORs are emerging as therapeutic targets for tumors, their roles in the modulation of host defense during infection in the context of autophagy are unclear. Upon infection with highly pathogenic avian influenza viruses (HPAIV H5N1), RORα is synthesized and suppresses NF-κB signaling and the inflammatory response in monocytes, thereby contributing to the escape of H5N1 from the host inflammatory defenses [182]. RORα is a melatonin receptor and protects against ischemic heart injury and diabetic cardiomyopathy [183,184]. The effect of melatonin on autophagy regulation has been reported in various normal and cancer cells [185,186]. In addition, the protective effects of melatonin in various bacterial, viral, and parasitic infections have been characterized [187,188,189,190,191]. Melatonin treatment of Hodgkin lymphoma cells increased the expression of RORα, RORβ, and RORγ, and enhanced autophagy activation [192]. Therefore, it would be interesting to investigate the involvement of RORs in melatonin-mediated autophagy activation.

### 8.4. Farnesoid X Receptors-α (FXR-α)

As a nutrient-sensing and autophagy-regulating NR, FXRα regulates hepatic autophagy to maintain the energy balance in the liver [22,193] and inhibits autophagy-mediated ciliogenesis [143]. The inhibitory effect of FXRα is counteracted by PPARα, which activates autophagy [22,143,193]. Because PPARα is involved in the coordination of autophagy activation and antimicrobial defenses [72], it would be interesting to investigate the role of FXRα.

In a model of cholestasis, the activation of autophagy maturation is inhibited in an FXR-dependent manner, partly as a result of the induction of Rubicon. However, ursodeoxycholic acid (UDCA), which is a non-FXR-agonistic bile acid, induced hepatic autophagy and reduced the expression of Rubicon, which is an inhibitor of autophagy [194]. In an autophagy-deficient liver, the expression of FXR and its downstream genes was inhibited, promoting cholestatic injury [195]. Therefore, the link between FXR and autophagy requires further investigation.

### 8.5. Liver X Receptor (LXR)-α (LXRα; NR1H3) and -β (LXRβ; NR1H2)

LXRα and LXRβ are negative regulators of cholesterol metabolism and inflammation [196,197]. Both LXRs are important in the antimicrobial response to viral and bacterial infections [75]. Three synthetic LXR agonists (T0901317, GW3965, and LXR-623) had a long-lasting inhibitory effect on hepatitis B virus replication and gene expression [198]. In addition, both LXRα and LXRβ were required for the suppression of gammaherpesvirus reactivation by downregulating fatty acid and cholesterol synthesis in macrophages [199], and for the inhibition of herpes simplex virus type 1 (HSV-1) by 25-hydroxycholesterol [200]. IL-36 and LXR signaling promoted anti-mycobacterial effects by inducing the expression of cholesterol-converting enzymes and regulating the expression of antimicrobial peptides [201]. In addition, LXR activation inhibited *Salmonella* infection by inducing the expression of the multifunctional enzyme CD38 [202]. LXRs are involved in the regulation of autophagy in various pathological conditions, including cancers [203,204]. Therefore, further studies on the involvement of LXRs in modulating autophagy during infection with intracellular microbes are required.

### 8.6. Thyroid Hormone Receptors-α (TRα; THRA; NR1A1) and -β (TRβ; THRB; NR1A2)

The thyroid hormone is a regulator of the metabolic rate, oxidative phosphorylation (OXPHOS), and ROS production [205,206], and a potent inducer of autophagy/mitophagy [207,208]. Thyroid hormone suppresses the hepatic carcinogenesis induced by the HBV X protein by promoting mitochondrial turnover via autophagy activation. In addition, thyroid hormone/TR-induced hepatic PINK1 expression is associated with hepatic cellular carcinoma (HCC) progression and a poor prognosis [209]. One might expect that thyroid hormone is involved in the modulation of host antimicrobial responses through autophagy. However, it remains to be determined whether both TRα and TRβ contribute to pathogenesis or protective immunity to broader ranges of infections through autophagy modulation. The studies on several NRs with potential roles in connecting autophagy and host defense are summarized in Table 4.

## 9. Conclusions

Given the role of autophagy in controlling intracellular pathogens, there is an urgent need for autophagy-directed therapeutics and prophylactics. The targeting of autophagy in monocytes/macrophages stimulates the innate immune response, the dampening of inflammation and suppression of innate immunity, and the promotion of pathogen escape [210]. Therefore, a more comprehensive understanding of the molecular mechanisms of crosstalk between autophagy and the innate immunity system in acute vs. chronic infection by various pathogens in immunocompetent vs. immunocompromised hosts will facilitate the development of therapeutics and vaccines.

NR protects against a variety of infections and modulates autophagy during pathogen invasion. Early studies exploited VDR- or ERRα-targeted antimicrobial responses and were followed by several trials expanding the effects of other NRs. It is known that the ATGs involved in non-canonical autophagy are different from those involved in canonical autophagy, but only a few studies on NR-mediated non-canonical autophagy pathways have been reported to date [211]. We are only beginning to answer important questions on the NR regulation of autophagy and innate immune responses. It is important to understand the signaling networks connecting NRs, autophagy, and the inflammatory and immune responses according to the infection stage and pathogen. Such an enhanced understanding will facilitate the development of novel antimicrobials.

## Figures and Tables

**Table 1:** Vitamin D receptor (VDR) in autophagy-mediated host defense against infections.

Ligand/Activator	Pathogen/Disease	Study Model	Autophagy	Effects	Mechanism of Action	Ref.
**Bacterial/Fungal infections**						
Vit-D	*H. pylori*	Human gastric epithelial cell lines, clinical specimens	↑	Bacterial eradication	Activation of PDIA3 receptor and nuclear translocation of PDIA3-STAT3 complex to induce autophagosomal degradation independent of VDR	[106]
	Mtb	Human primary monocytes, MDMs, THP-1, and RAW264.7 cells	↑	Antimicrobial	Beclin-1 and Atg-5 activation mediated through hCAP-18/LL-37-dependent MAPK and C/EBPβ activation	[107]
		PGE2, human macrophages	↓	Intracellular Mtb survival	PGE2 inhibits hCAP18/LL-37 expression and vitamin D-induced cathelicidin and autophagy by dampening expression of VDR	[114]
		Mouse model	↑	Antimicrobial	Vit-D supplementation on 2nd-line anti-TB therapy leads to suppression of MMP1 Induction of VDR, CRAMP, LC3B, and caspase-3	[115]
		IFN-γ, human macrophages	↑	Antimicrobial	Vitamin D-dependent autophagy and autophagolysosomal fusion by IFN-γ VDR-dependent induction of cathelicidin and DEFB4 by IFN-γ	[121]
	*A. fumigatus*	Alveolar macrophages	↓	Antimicrobial	Delayed formation of lysosomes against infection Modulation of Dectin-1, ROS, and LC3 expression	[127]
	*M. marinum*	Human monocytes (THP-1)	↑	Antibacterial	Induction of endogenous CAMP and its colocalization with autophagolysosome	[110]
Vit-D-sufficient human serum	Mtb	CD40L, IFN-γ, human PBMC	↑	Antimicrobial	Induction of CYP27B1, VDR, cathelicidin, and DEFB4	[122]
**-**	Mtb lipoprotein LpqH	Human primary monocytes	↑	Antibacterial	TLR2/1/CD14-mediated (C/EBP)-β-dependent induction of CYP27B1	[109]
PBA+Vit-D	Mtb	PBMCs and MDMs from TB patients	↑	Antimicrobial	Increased LC3 expression, decreased *XBP1spl* mRNA	[116]
	TB patients	Clinical trial	-	Antimicrobial	Increased LL-37	[117]
		Clinical trial	-	Clinical recovery	Increased serum Vit-D levels after PBA+Vit-D supplementation	[118]
LAB	*S. enterica*	HCT116, MEFs cell lines, in vivo mice	↑	Anti-inflammatory	Enhanced expression of Beclin-1 and ATG16L1 Increased expression of VDR and cathelicidin	[126]
**Viral infections**						
Vit-D	HIV	Human MDMs	↑	Inhibition of virus replication	PI3K-, ATG-5-, and Beclin-1-dependent autophagy activation	[111]
	HIV, Mtb	Human MDMs	↑	Inhibition of virus replication and mycobacterial growth	Induced expression of CAMP	[112]
	Influenza A	A549 cell lines	↑	Antiviral	Restoration of virus-induced inhibition of autophagic flux through Syntaxin-17 and ATP6V0A2	[125]
	Rotavirus	Pigs, IPEC-J2 cells	↑	Inhibition of virus infection	Regulation of autophagic maturation and expression of porcine cathelicidin genes	[128]
TLR8 ligands	HIV	Human macrophages	↑	Reduced virus replication	Induced expression of CAMP, VDR, and CYP27B1	[113]

Vit-D, 1,25-dihydroxy vitamin D3; PDIA3, protein disulfide-isomerase A3; STAT3, signal transducer and activator of transcription 3; Mtb, *Mycobacterium tuberculosis*; MDM, monocyte-derived macrophages; hCAP-18, human cationic antimicrobial protein; MAPK, mitogen-activated protein kinase; C/EBPβ, CCAAT/enhancer-binding protein beta; CAMP, cathelicidin antimicrobial peptides; TLR, toll-like receptor; PGE2, prostaglandin E2; PBA, phenylbutyrate; XBP1, X-box binding protein 1; TB, tuberculosis; IFN, interferon; DEFB4, beta-defensin 2; PBMC, peripheral blood mononuclear cells; CYP27B1, cytochrome p450 27B1 or 25-Hydroxyvitamin D3 1-α-hydroxylase; LAB, lactic acid bacteria; MEF, mouse embryonic fibroblasts; HCT116, human colon cancer cell line; ATG16L1, autophagy related 16 like 1; ROS, reactive oxygen species; HIV, human immunodeficiency virus; PI3K, phosphoinositide 3-kinases; ATP6V0A2, V-ATPase 116 kDa isoform a2.

**Table 2:** Estrogen receptors (ERs)/estrogen-related receptors (ESRRs).

NRs	Ligands/Activator	Pathogen/Study Model	Autophagy	Effects	Mechanism of Action	Ref.
ER	Estrogen (E2)	Thyroid cancer patients samples, Nthy-ori 3-1, PTC cell, BCPAP-ERα, MCF-7 cells	↑	Tumor cell survival	Generation of ROS, activation of ERK1/2	[131]
	Bazedoxifene	Mtb/THP-1 cells	↑	Inhibition of intracellular growth of Mtb	Increased ROS and phosphorylation of Akt/mTOR signaling	[90]
ERRα	AICAR	Mtb/BMDMs, RAW264.7, HEK293T cells	↑	Antimicrobial host defense	Transcriptional activation of autophagy-related genes, and post transcriptional activation of autophagy through SIRT1 activation	[23]
	Thyroid hormone	THRB1-HepG2 cells, in vivo mice model	-	Mitophagy induction	Increased ESRRA expression via PPARGC1A Induction of ULK1 mRNA and protein through ESRRA-dependent transcription	[138]
	XCT 790	SH-SY5Y, HeLa cells, in vivo mice	↑	Neuroprotective	Regulation of autophagy by ERRα through its localization with autophagosome	[139]

PTC, papillary thyroid carcinoma; MCF-7, breast cancer cell line; ERK1/2, extracellular signal-regulated protein kinase; mTOR, mammalian target of rapamycin; AICAR, 5-aminoimidazole-4-carboxamide ribonucleotide; BMDM, bone marrow-derived macrophages; SIRT1, sirtuin 1; PPARGC1A, peroxisome proliferator-activated receptor gamma coactivator 1-alpha; ULK1, unc-51 like autophagy activating kinase.

**Table 3:** Peroxisome proliferator-activated receptors (PPARs).

NRs	Ligands/Activator	Pathogen	Pathogen/Study Model	Autophagy	Effects	Mechanism of Action	Ref.
PPARα	GW7647, Wy14643	Mtb	BMDMs, in vivo mice	↑	Antimicrobial	Increased expression and nuclear translocation of TFEB	[72]
	Gemfibrozil	*M. abscessus*	BMDMs, in vivo mice	↑	Antimicrobial	Increased nuclear translocation of TFEB	[70]
PPARβ/δ	GW501516	-	Human cardiac AC16 cells, in vivo mice	↑	Inhibition of palmitate induced ER stress	Upregulation of Beclin-1 and LC3II	[155]
PPARγ	HP24	*T. cruzi*	Peritoneal macrophages, in vivo mice	-	Pro-angiogenic and anti-inflammatory	Induction of pro-angiogenic mediators (eNOS and VEGF-A) through PI3K/Akt/mTOR and PPARγ pathway Inhibition of NF-κB pathway in PPARγ-dependent manner	[159]
	INT131	EcoHIV	Primary mouse glial cells, in vivo mice	-	Anti-inflammatory	Inhibition of proinflammatory cytokines	[160]

TFEB, transcription factor EB; eNOS, endothelial NOS; VEGF-A, vascular endothelial growth factor A; NF-κB, nuclear factor kappa-light-chain-enhancer of activated B cells.

**Table 4:** Several nuclear receptors (NRs) with potential functions in linking autophagy and host defense.

NRs	Ligands/Activator	Pathogen/Study Model	Autophagy	Effects	Mechanism of Action	Ref.
REV-ERBα	GSK4122	Zebrafish	Rhythmic with circadian clock	Regulation of autophagy rhythms	Direct regulation of NR1D1 and CEBPB by nutritional signals and circadian clock	[164]
	-	Mouse model, MEF, Hepa1-6 and HEK293 cells	Rhythmic with circadian clock	Regulation of autophagy rhythms	Autophagy activation through TFEB and TFE3; repression by REV-ERBα	[162]
	GSK4112	Mtb/Human macrophages	↑	Antimicrobial	Modulation of LAMP1 and TFEB, repression of IL10	[165]
	SR9009, SR9011	Cancer cell lines, human glioblastoma stem cells, in vivo mice	↓	Anticancer	REV-ERB agonist inhibit autophagy (decreased LC3, increased p62, and increased LAMP1) and de novo lipogenesis to induce apoptotic responses	[163]
		in vivo mice, C2C12 myoblasts	↓	Improved muscle oxidative function	LKB1-AMPK-SIRT1–PPARGC1A signaling pathway	[167]
RARα	ATRA	HeLa, APL NB4 cells	↑	Differentiation of APL cells	Inhibition of mTOR pathway to induce autophagy-dependent PML/RARA degradation	[171]
		APL patients samples, NB4 cell lines	↑	Differentiation of APL cells	MIR125B1 overexpression enhanced PML-RARA expression DRAM2 as a target of MIR125B1	[172]
RORα	-	HPAIV (H5N1)/human monocytes,	-	Inhibition of inflammatory responses	H5N1 inhibits NF-κB and activates RORα in monocytes	[182]
	-	MI/R injury mice model	↑ or preservation of autophagy function	Protection against MI/R injury	Inhibition of ER stress and mitochondrial apoptosis pathway, restoration of autophagy function, reduced oxidative/nitrative stress	[183]
RORγ	Melatonin	Human HL cell line L428	↑	Cell death	Induction of autophagic cell death by melatonin via increased level of RORC	[192]
FXR/PPARα	GW4064/GW7647	Mouse primary hepatocytes, mouse liver	FXR: ↓, PPAR: ↑	-	FXR and PPAR compete for binding to common sites in autophagic gene promotors, with opposite transcriptional outputs	[22]
	GW4064/GW7646, Wy14643	Human RPE cells, MEFs, HK2, A549 cells	FXR: ↓, PPAR: ↑	FXR represses and PPAR facilitates cliogenesis	Regulation of expression of autophagic genes, FXR acts in opposite way with PPARA	[143]
FXR	Bile acids	Liver tissue from cholestasis patients, HepG2 cells	↓	Autophagy and Rubicon could be novel treatment target for cholestatic liver disease	Prevention of proper fusion of autophagolysosome with lysosomes by bile acids, through FXR-dependent induction of Rubicon	[194]
	GW4064	In vivo mice with hepatic deletion of *Atg7* or *Atg5* with or without *Nrf2* codeletion	↓	Liver injury	NRF2 activation in autophagy deficiency leading to downregulation of FXR, causing cholestasis	[195]
LXR	T0901317, GW3965, LXR-623	HBV/primary human hepatocytes, HepaRG cells	-	Anti-HBV effects	Inhibition of cholesterol 7α-hydroxylase 1 (CYP7A1) mRNA levels	[198]
	DDA	Melanoma and AML cell lines, AML patients samples, in vivo mice	↑	Anti-tumor	DDA acting as partial agonist on LRX to increase Nur77, Nor1, and LC3 expression	[204]
TR	T3	HepG2, Huh7 cells	↑	Lipid metabolism	Upregulation of C19orf80 expression, which is involved in lipid metabolism through breakdown of lipid droplets	[208]
		Mice model of hepatocarcinogenesis, HepG2 cells	↑	Inhibition of hepatic DNA damage, inflammation, and carcinogenesis	Induction of hepatic PINK1 expression, which ubiquitinates HBx protein to trigger mitophagy	[209]

CEBPB, CCAAT/enhancer-binding protein beta; TFEB, transcription factor EB; TFE3, transcription factor E3; LAMP1, lysosomal-associated membrane protein 1; IL, interleukin; LKB1, liver kinase B1; AMPK, AMP-activated protein kinase; DRAM2, DNA-damage regulated autophagy modulator; PML, promyelocytic leukemia; ATRA, all-trans-retinoic acid; HPAIV, highly pathogenic avian influenza virus; APL, acute promyelocytic leukemia; MI/R, myocardial ischemia/reperfusion; HL, Hodgkin lymphoma; RPE, retinal pigment epithelium cells; NRF2, nuclear factor erythroid 2-related factor 2; HBV, hepatitis B virus; DDA, dendrogenin A; Nur77, nerve growth factor IB; Nor1, neuron-derived orphan receptor 1; T3, triiodothyronine; PINK1, PTEN-induced kinase 1.
